# Prosthetic treatment of proximal humerus fractures in the elderly

**DOI:** 10.1007/s00068-025-02867-x

**Published:** 2025-05-12

**Authors:** René D. Verboket, Klaus W. Wendt, Maren Janko, Ingo Marzi

**Affiliations:** 1 Department of Trauma Surgery and Orthopedics, Goethe University Frankfurt, University Hospital, Frankfurt, Germany; 2 Trauma Surgery, University Medical Center Groningen, Groningen, Netherlands

## Abstract

The Section for Skeletal Trauma and Sports Injuries of the European Society for Trauma and Emergency Surgery (ESTES) has reached a consensus among European countries in the treatment of proximal humerus fractures in a working group. As a result the ESTES recommendations on proximal humerus fractures in the elderly were published in 2021. The various treatment options and algorithms for this are now described in more detail and the procedures were explained in several related specialist articles. The recommendations include conservative and four possible surgical treatment options (ORIF, nailing, hemi- and total inverse arthroplasty). This article deals with hemi- and total inverse arthroplasty. Reverse total shoulder arthroplasty (RTSA) has emerged as a key treatment for complex proximal humerus fractures in elderly patients. It offers significant advantages in restoring function, alleviating pain, and providing durable outcomes compared to alternative approaches. Despite challenges related to implant complications and surgical expertise, ongoing advancements in technology and techniques continue to improve its effectiveness. With the rising incidence of complex fractures, RTSA is expected to play an increasingly vital role in maintaining the quality of life in aging populations.

## Introduction

Proximal humerus fractures are among the most common injuries in elderly patients, accounting for approximately 6% of all fractures [1]. Their incidence rises significantly with age, primarily due to osteoporosis and increased fall risk. These fractures are often caused by low-energy trauma, such as falls from standing height. With an aging population, the prevalence of these injuries is projected to triple by 2030, posing significant challenges for healthcare systems globally [2–4]. Effective and sustainable treatment options are crucial to address this growing burden, particularly for elderly patients who often have diminished bone quality and comorbidities that complicate recovery [5].

The management of proximal humerus fractures in older adults presents unique difficulties [6]. Osteoporotic bone structure frequently limits the effectiveness of traditional fixation methods, such as open reduction and internal fixation (ORIF), due to a higher risk of implant failure or secondary fracture displacement [7–11]. Complex three- or four-part fractures are particularly problematic, often resulting in poor functional outcomes when treated non-operatively [12]. While conservative approaches may suffice for minimally displaced fractures [13, 14], displaced or comminuted fractures with vascular compromise of the humeral head frequently necessitate surgical intervention [15].

Key issues in the treatment include poor bone quality, often due to osteoporosis [16–19], and the complexity of managing displaced, multifragmentary fractures of the tuberosities [20–22]. Additional difficulties arise with 4-part fracture dislocations, 4-part fractures with a thin and displaced humeral head segment, and varus displaced fractures [21–23]. Particularly problematic are head split fractures and injuries with a high risk of ischemia, as determined by Hertel’s criteria [24].

These fracture types generally respond poorly to non-operative treatment, showing limited potential for healing and functional recovery [21, 23, 24]. In such scenarios, a shoulder replacement—either hemiarthroplasty or reverse total shoulder arthroplasty—may be necessary to achieve acceptable functional outcomes and restore quality of life for the patient [6, 25].

Prosthetic solutions, including hemiarthroplasty and reverse total shoulder arthroplasty (RTSA), have become important in the surgical treatment of complex proximal humerus fractures in elderly patients [26]. These approaches aim to restore shoulder function and alleviate pain, with the choice of prosthesis depending on factors such as fracture pattern, rotator cuff integrity, and patient-specific functional demands.

**Hemiarthroplasty (HA)** has historically been a popular option for treating proximal humerus fractures. In this procedure, the humeral head is replaced with a prosthetic implant while retaining the patient’s glenoid. This approach is often selected for fractures where the rotator cuff remains intact, allowing for restoration of humeral head stability and shoulder biomechanics [26]. However, long-term outcomes of hemiarthroplasty have been mixed [27]. Functional results heavily depend on anatomical reconstruction and proper tuberosity fixation [28], which can be challenging in osteoporotic bone [29]. Studies have shown that patients often experience limited range of motion and suboptimal functional recovery, particularly if tuberosity healing fails or if the rotator cuff becomes dysfunctional over time [30, 31]. Younger and more active patients with preserved rotator cuff function may still benefit from hemiarthroplasty, provided that the fracture pattern and bone quality allow for reliable tuberosity healing [32]. Nonetheless, this subset of patients is becoming increasingly rare in the context of osteoporotic fractures, particularly in those over 70 years of age.

**Reverse total shoulder arthroplasty (RTSA)** has emerged as the preferred prosthetic option for elderly patients with complex fractures, particularly in cases with irreparable rotator cuff tears or significant tuberosity comminution [33]. This design reverses the natural anatomy of the shoulder joint by placing a convex ball on the glenoid and a concave socket on the humerus. By shifting the center of rotation medially and inferiorly, RTSA optimizes the function of the deltoid muscle, compensating for the loss of rotator cuff function. RTSA provides reliable pain relief and substantial improvements in range of motion, particularly in forward elevation, making it a superior choice for older adults with high functional demands [27, 34–36].

Longitudinal studies have demonstrated the durability of RTSA outcomes, with significant functional improvements maintained for up to 20 years postoperatively [37]. However, the procedure is not without complications, such as scapular notching, implant loosening, and limited external rotation. These risks underline the importance of meticulous surgical planning and patient selection. RTSA is generally recommended for elderly patients with severe comminution, pre-existing shoulder arthritis, or poor bone quality that precludes effective fixation [6].

As the demand for prosthetic solutions grows, advancements in prosthetic design, surgical techniques, and perioperative care are crucial to further improving outcomes. Innovations such as bioactive materials, patient-specific implants, and minimally invasive approaches hold promise for enhancing the effectiveness and safety of prosthetic shoulder replacements. A multidisciplinary approach, integrating geriatric assessment, physical therapy, and fall prevention strategies, is essential to maximize recovery and maintain independence for elderly patients [26, 30].

**Fig. 1:**
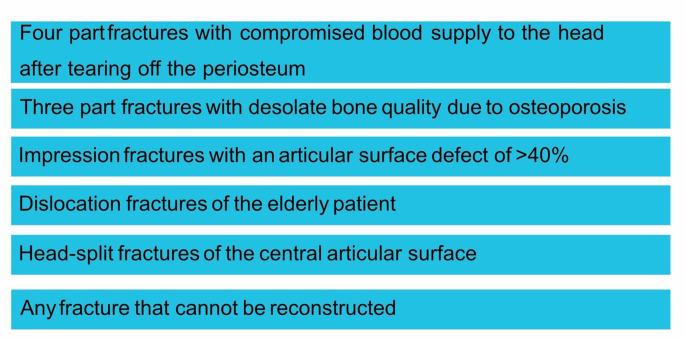
: Possible indication criteria for the primary implantation of an inverse shoulder endoprosthesis (Frank, 2020) [38]

This work was carried out in order to show the current state of research in the treatment of proximal humerus fractures in elderly patients and to help to find a tailored approach. While hemiarthroplasty remains a viable option in select cases, RTSA has become the gold standard for complex fractures in this population (Fig. 1). By addressing the unique challenges of aging patients, prosthetic interventions play a pivotal role in preserving mobility, reducing pain, and improving the overall quality of life.

## Surgical technique reverse total shoulder arthroplasty (RTSA)

Reverse total shoulder arthroplasty (RTSA) has become a cornerstone in treating complex proximal humerus fractures, massive irreparable rotator cuff tears, and cuff tear arthropathy, particularly in elderly patients [6, 26, 27]. This procedure involves reversing the natural anatomy of the shoulder joint to optimize deltoid function, compensating for deficient rotator cuff mechanics. A detailed understanding of the surgical steps and precise execution are essential to achieve optimal outcomes and reduce complications.

The procedure begins with positioning the patient in the beach-chair position on a radiolucent table, allowing easy access to the shoulder and facilitating intraoperative imaging. The most commonly used approach is the deltopectoral approach, which provides excellent visualization of the glenoid and humeral structures while minimizing soft tissue damage. Alternatively, a deltoid-splitting approach may be utilized based on specific anatomical considerations, however, this approach has a higher risk for the axillary nerve, which is outmost important for the success of the RTSA [39].

**Fig. 2:**
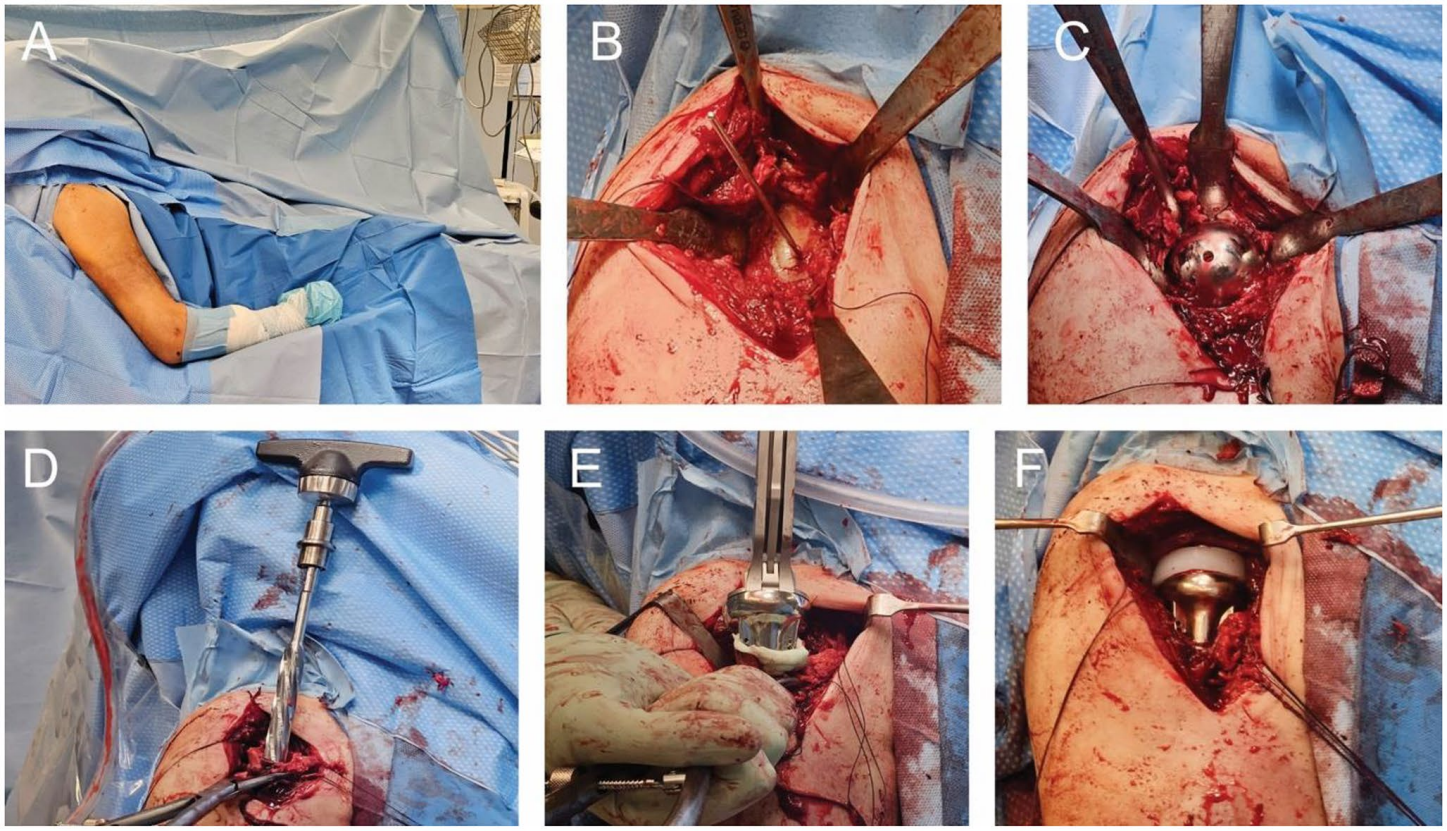
Implantation of a reverse shoulder endoprosthesis. **A**: Positioning in the beach chair position; **B**: Deltopectoral approach with exposure of the glenoid and securing of the rotator cuff; **C**: Implantation of the glenosphere, the entry point is thus selected so that the metaglene is flush with the lower bony glenoid; **D**: Preparation of the humeral shaft; **E**: Cemented implantation of the humeral stem; **F**: Insertion of the inlay, followed by closure of the rotator cuff with possible fixation to the prosthetic stem

After incising the skin and exposing the joint, the humeral head is resected, often including fractured or necrotic segments. Particular care is taken to preserve the tuberosities, as their reattachment significantly influences postoperative function. After careful dissection of the tuberosities using a tenotomy of the supraspinatus tendon in the direction of the fibers, the tuberosities are backstitched and reinforced with strong sutures (e.g. FibreWire) in the area of the tendon insertions. Once the head fragment has been salvaged, it is used as a template for the diameter and height of the future head of the prosthesis. The glenoid is then initially prepared in the form of an extensive release of the soft tissue. The risk of dislocation is increased by too little soft tissue release rather than too much. The capsule and labrum are then completely resected. A guide wire for the metaglene drill guide is inserted inferior and slightly posterior to the center point at a maximum declination of 5–10°. The entry point is thus selected so that the metaglene is flush with the lower bony glenoid (See Fig. 2).

The existing cartilage is then removed over the guide wire using a reamer, a central drill hole is created and the guide pin of the metaglene is inserted over it. This is attached using four screws, both cortical and locking screws are available depending on bone quality. The upper and lower screws should be implanted at a slightly ascending and descending angle. The glenosphere can now be placed in the appropriate size. After tightening the screw, the glenosphere should be retightened after tapping it with a hammer, as it may tighten further. The humeral shaft can then be prepared. To do this, the medullary cavity is prepared with the awl or a rasp after exposing the stem (through external rotation, adduction and retroversion) and the appropriate prosthesis size is determined. Depending on the prosthesis system, the stem can be cemented or cementless. The alignment of the socket is planned with a retroversion of 0–20°. This should be meticulously adjusted. A trial socket and a trial inlay are then fitted and the prosthesis is repositioned to check the correct soft tissue tension, mobility and alignment. The tension is checked by longitudinal traction.

**Fig. 3:**
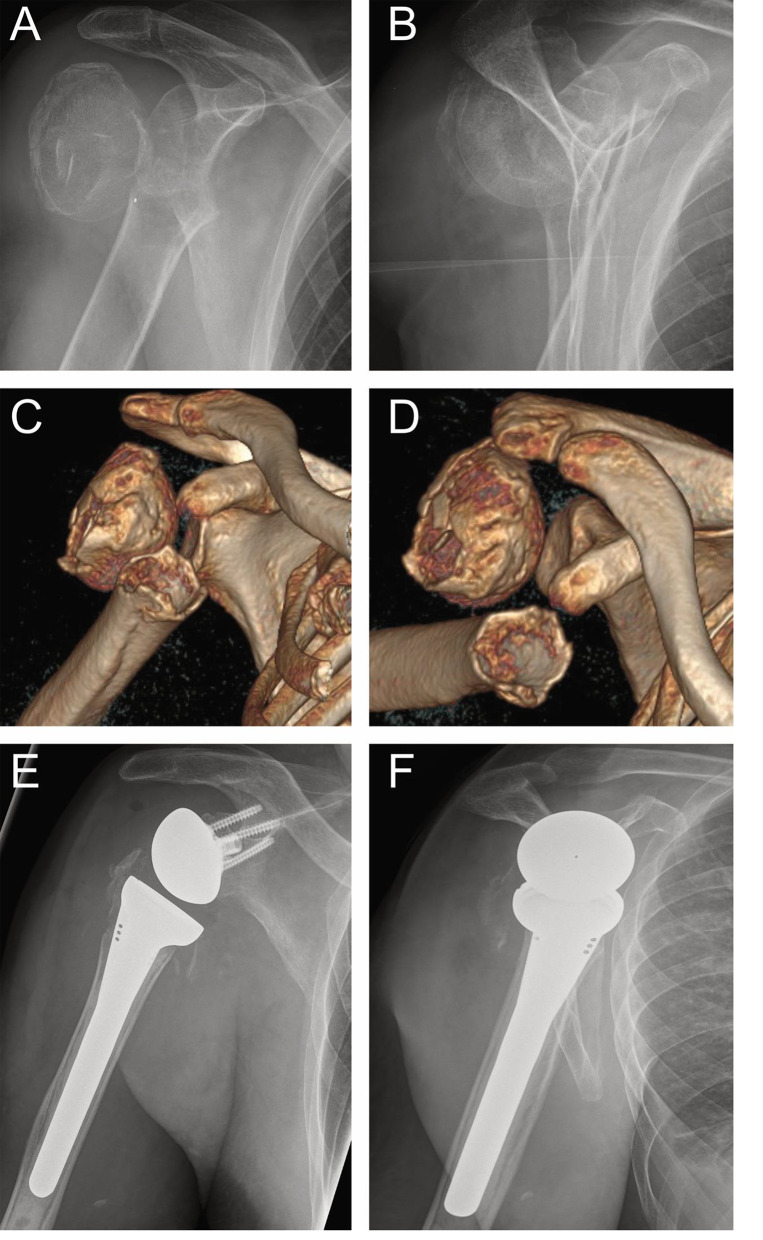
Preoperative a.p. (**A**) and y-view (**B**) X-ray of a complete head dislocation in an elderly patient, **C**, **D** 3D reconstruction of the fracture with head split component, and after surgical treatment with inverse shoulder endoprosthesis as a.p. X-ray (**E**) and y-view X-ray (**F**)

On the humeral side, a metaphyseal component with a concave polyethylene liner is selected and positioned based on the anatomy and bone quality. In elderly patients with osteoporotic bone, cement fixation may be considered. The humeral stem is implanted into the prepared canal, and the liner is fixed into place (See Fig. 3).

**Fig. 4:**
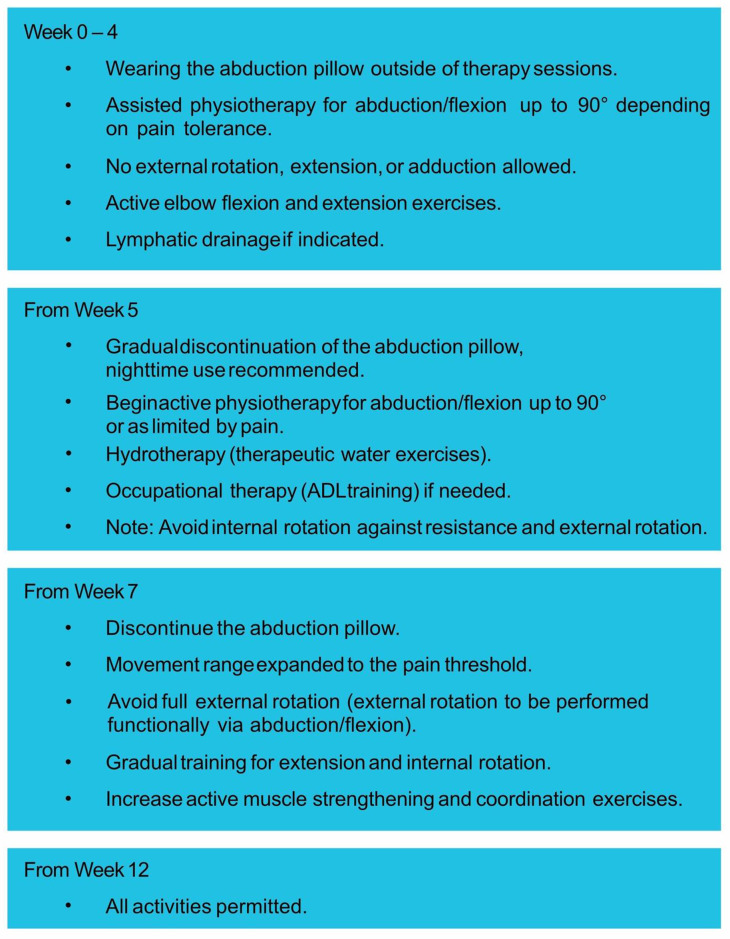
Example for post-operative treatment program

If the tension is too loose, the prosthesis will dislocate. In the latter case, for example, higher inlays or a larger glenosphere can be used to increase the tension. Spacers are also available if more distance needs to be compensated. After implantation of the definitive prosthesis, all refixable and reconstructable parts of the rotator cuff should be tied to the prosthesis using non-absorbable, tear-resistant sutures (e.g. FibreWire). This improves the muscular guidance of the prosthesis. The aim is early functional postoperative mobilization. Accordingly, an abduction pillow should be applied for at least 4–6 weeks postoperatively and gradually removed from the fifth week onwards. During the first four weeks, physiotherapy exercises are carried out with assistance while limiting abduction/flexion to 90°. External rotation, extension and adduction should be avoided during this time. From the fifth week, active exercise of the shoulder up to the horizontal begins. External rotation should be avoided for a full 6 weeks. Only from the 12th postoperative week is the full range of motion and full weight-bearing allowed without limitation. The definitive post-operative treatment program depends on the surgeon and clinic (see Fig. 4).

Rehabilitation follows a phased protocol, beginning with passive and assisted range-of-motion exercises and progressing to active strengthening as healing allows. The ultimate goal is to restore functional range of motion and pain-free shoulder use. Long-term follow-up is crucial to monitor for complications such as scapular notching, implant loosening, or limited external rotation.

### Possible complications

**Fig. 5:**
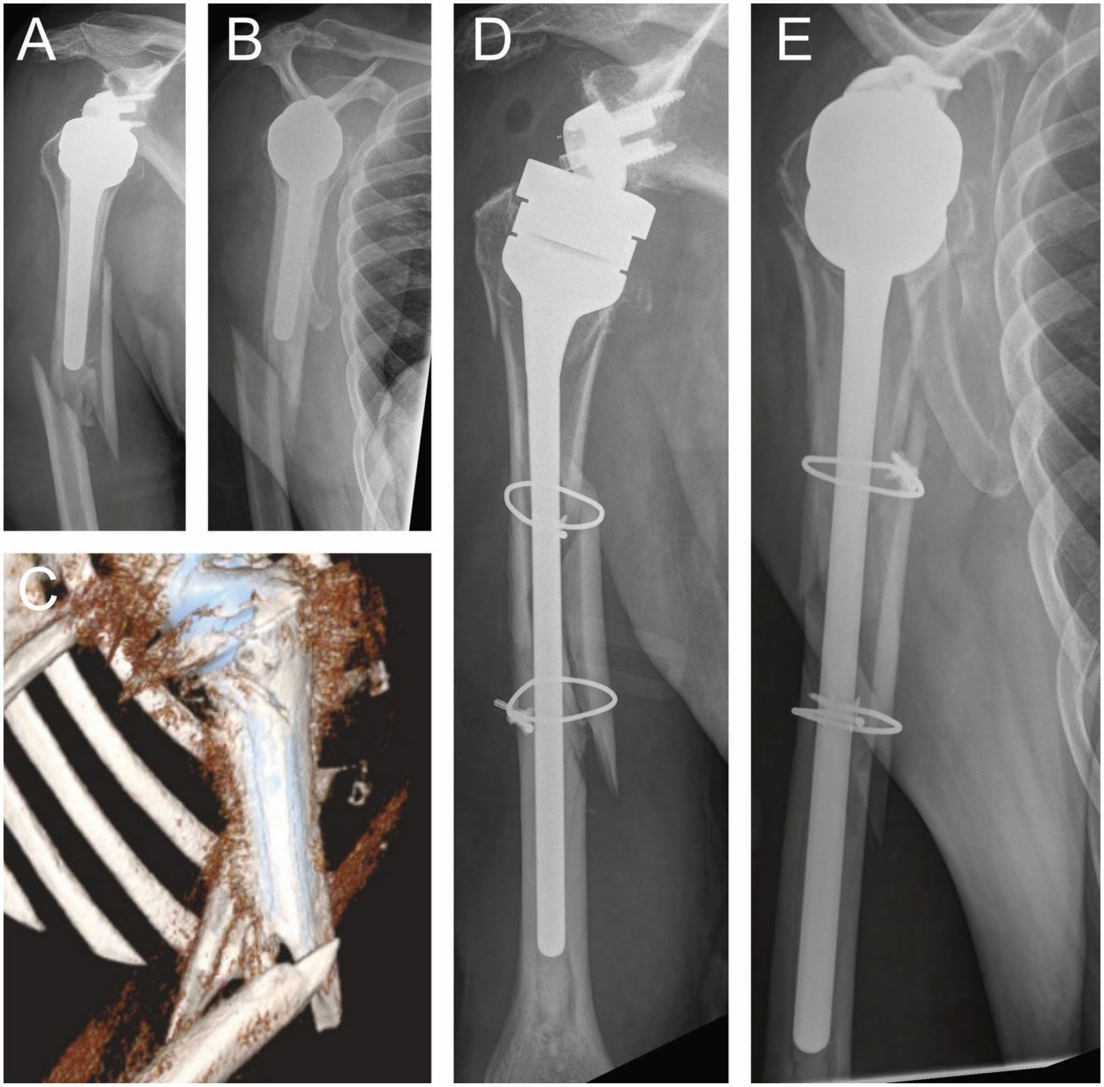
Preoperative a.p. and y-view X-ray with periprothetic fracture (**A**, **B**), 3D reconstruction in CT image auf the fracture C, and after surgical treatment with stem change inverse shoulder endoprosthesis a.p. X-ray and y-view X-ray (**D**, **E**)

Although reverse shoulder arthroplasty (RTSA) is a highly effective procedure for treating complex shoulder pathologies, it is not without potential complications. One common issue is scapular notching, which occurs when the humeral component contacts the scapular neck during movement, potentially leading to implant wear and reduced range of motion. To prevent scapular notching, an eccentric glenosphere can be selected, along with specialized components that increase the humeral head’s lateral offset. Glenoid component loosening is another concern, particularly in cases of poor bone quality or improper fixation, which can result in implant instability [27, 34]. Infection, while rare, can lead to significant morbidity and may necessitate revision surgery. Other complications include nerve injuries, such as axillary nerve damage, which can impair deltoid function, and humeral fractures, particularly in osteoporotic bone during stem placement. Patients may also experience limited external rotation, often due to the altered biomechanics of the prosthesis or soft tissue tensioning. Additionally, hematoma formation and shoulder stiffness can occur, especially if postoperative rehabilitation is delayed [30, 37]. Early identification and management of these complications are critical to ensure optimal functional outcomes and patient satisfaction. As longer term complication periprosthetic fractures occur and need to be treated either with cerclage wires or in most cases with a revision stem or both in combination (see Fig. 5). Studies on the mid-term outcomes of RTSA have reported a decline in the Constant-Murley score after 6 to 8 years, primarily due to a loss of forward elevation and strength. This has been attributed to increased stress on the deltoid muscle, potentially leading to “deltoid fatigue.” A gradual reduction in overhead range of motion (ROM), averaging 0.8° per year, was identified which occurs at a faster rate in RTSA than in the natural shoulder. This progressive deterioration was found to be independent of age, gender, and preoperative diagnosis [40].

## Discussion

Proximal humerus fractures are among the most common injuries in elderly patients, often resulting from low-energy trauma such as falls from standing height. These fractures disproportionately affect individuals over 60 years of age due to the prevalence of osteoporosis and reduced bone quality, with three- and four-part fractures being particularly frequent in this demographic [14, 20, 25]. The management of these injuries poses significant challenges due to the complexity of fracture patterns, diminished bone healing capacity, and the presence of comorbidities [6].

Prosthetic treatment, particularly reverse total shoulder arthroplasty (RTSA), has emerged as a pivotal approach for addressing complex proximal humerus fractures in elderly patients [41, 42]. Traditional treatment options, such as conservative management and open reduction with internal fixation (ORIF), often fail to provide satisfactory outcomes in cases of severe displacement, avascular necrosis, or poor bone quality. Prosthetic solutions are increasingly favored for their ability to restore function and alleviate pain, especially in patients with compromised rotator cuff integrity [13, 21, 41, 43].

Several studies have reported that hemi arthroplasty (HA) provides acceptable pain relief; however, functional outcomes are often compromised by displacement, nonunion, or resorption of the tuberosities. It is widely recognized that anatomical healing of the tuberosities is a key factor for successful HA, as it is essential for rotator cuff recovery. The likelihood of HA failure increases in cases of fracture comminution, osteopenia, impaired vascularization, or rotator cuff insufficiency—conditions commonly seen in elderly patients.

Reverse total shoulder arthroplasty (RTSA) is a semi-constrained prosthesis that allows the deltoid muscle to compensate for a deficient rotator cuff. By establishing a stable center of rotation in the glenoid, RTSA enables active arm flexion and abduction. Although the positioning and healing of the tuberosities also influence RTSA outcomes—particularly in restoring external rotation—their impact is less critical compared to HA.

The literature comparing HA and RTSA outcomes is not conclusive. Some studies indicate that RTSA results in superior functional outcomes, while others report no significant difference. Numerous variables influence the success of shoulder arthroplasty for fractures.

### Advantages of reverse total shoulder arthroplasty

RTSA represents a paradigm shift in the management of proximal humerus fractures, especially those involving three- or four-part fractures where rotator cuff dysfunction limits traditional surgical efficacy [23, 24, 28]. This technique leverages altered shoulder biomechanics by reversing the normal ball-and-socket configuration, placing the convex component on the scapula and the concave component on the humerus. This configuration enhances the function of the deltoid muscle, compensating for the loss of rotator cuff stability. As a result, RTSA provides reliable pain relief, improved range of motion (ROM), and significant functional recovery [44–48].

### Clinical outcomes and longevity

Long-term outcomes of RTSA are highly favorable. Studies have demonstrated sustained improvements in clinical scores such as the Constant score and the Subjective Shoulder Value over a long follow-up period (see Table 1). Patients achieve significant gains in active anterior elevation and abduction, with stable results even in advanced age.

**Table 1:** Clinical outcome and complication rate of the recent studies investigating reverse total shoulder arthroplasty (RTSA) in elderly (70+). RCS– retrospective cohort study, RCT– randomized controlled trail, PCS– prospective cohort study, PHF– proximal humeral fracture

Study	Design	mean age	Number RTSA	Fracture Type	Scores (mean)	Complications
Batar 2020 [49]	RCS	73 ± 6.4	33	Neer type 3/4	Constant score 49	13%
Baudi 2014 [34]	RCS	77	25	Four-part PHF with varus/ valgus fracture/humeral head dislocation	Constant score 56.2	0%
Bonnevialle 2016 [33]	RCS	78 ± 5	41	Four-part PHF	Constant score 57	10%
Boyer 2017 [50]	PCS	78 (66 to 91)	65	Three and four-part PHF	Constant score 77.6	14.9%
Bufquin 2007 [44]	RCS	78 (65 to 97)	43	Three and four-part PHF	Constant score 44	29%
Cazeneuve 2010 [45]	RCS	75 (58 to 92)	36	Three and four-part PHF	Constant score 53	19.4%
Chalmers 2014 [51]	RCS	77 ± 6	9	Three and four-part PHF	SST Score 7 ± 2	11.1%
Cuff 2013 [27]	PCS	74.8 (70 to 86)	27	Three and four-part PHF	SST Score 7.4	8%
Grubhofer 2016 [46]	RCS	77 (58–89)	52	Three and four-part PHF	Constant score 62	Revision rate stated only– 5%
Jonsson 2021 [52]	RCT	80.4 ± 4.5	41	Three and four-part PHF	Constant score 58.7	7.3%
Klein 2008 [47]	RCS	74.85 +/- 5.73	20	OTA Type B2, C2, C3	Constant score 67.9	15%
Laas 2021 [53]	RCT	74.8 (65–92)	17	Three and four-part PHF	Constant score 51	not stated
Repetto 2017 [54]	RCS	71.2 ± 7.5	27	Three and four-part PHF	Constant score 58.8	11.1%
Sebastia-Forcada 2014 [55]	RCT	74.7 (70–85)	31	Three and four-part PHF	Constant score 56.1	6.4%
Solomon 2016 [56]	RCS	77 (65–88)	16	Complex PHF	ASES Score 79	12.5%
Villodre-Jimenez 2016 [48]	RCS	74,9	30	Three and four-part PHF	Abbreviated Constant Score– 49.1	13.3%
Yahuaca 2020 [25]	RCS	73 ± 9	106	Neer type 2/3/4	only range of motion stated	15%

However, complications such as scapular notching, a phenomenon resulting from impingement between the humeral implant and the scapula, are frequently observed. Despite the high incidence of this finding—up to 29% in implants followed for over 10 years—it does not appear to significantly impair clinical outcomes. Hanisch et al. [57] reported that patients under 65 undergoing RTSA do not have a higher risk of complications, revisions, or adverse outcomes compared to older patients.

### Indications for RTSA

RTSA is primarily indicated for elderly patients with complex fractures that are unlikely to heal satisfactorily with conservative or reconstructive approaches. These include displaced three- and four-part fractures, fractures involving significant humeral head splitting, and cases of avascular necrosis. Additionally, RTSA is often the treatment of choice when pre-existing rotator cuff arthropathy is present, as this condition exacerbates the functional limitations of traditional interventions.

In elderly patients, the decision to proceed with RTSA must consider multiple factors, including biological age, bone quality, comorbidities, and the patient’s functional expectations. The procedure is particularly advantageous for individuals aiming to maintain independence and quality of life, as it offers predictable functional outcomes.

Comparison to Hemiarthroplasty.

Hemiarthroplasty (HA), which replaces only the humeral head, was once a standard treatment for severe proximal humerus fractures. While it provides effective pain relief, it often fails to restore shoulder function adequately, particularly in the presence of rotator cuff deficiencies. In contrast, RTSA addresses both pain and function, offering superior long-term outcomes. The biomechanical advantages of RTSA ensure that even patients with poor bone stock and complex fracture patterns can achieve significant functional improvements. Similarly, Kany et al. [58] found no significant difference in postoperative function between RTSA and anatomic TSA in patients under 50. Additionally, RTSA showed significantly lower complication and revision rates than HA, although postoperative function was poorer with a metal head compared to a carbon head. In contrast, Aibinder et al. [39] suggested that younger patients may face a higher risk of complications and revisions in RTSA, likely due to their increased activity levels.

Surgical Considerations and Rehabilitation.

Successful RTSA requires precise surgical planning and execution. Factors such as implant positioning, restoration of the deltoid’s tension, and reconstruction of the medial support are critical for optimizing outcomes. Advanced imaging techniques, including CT scans, are often utilized for preoperative planning to assess fracture patterns and guide implant selection.

Postoperative rehabilitation is equally essential to achieving favorable results. Early physiotherapy focuses on restoring ROM and minimizing stiffness, with progressive strengthening exercises introduced as healing progresses. The ability to initiate early mobilization, a key advantage of RTSA, minimizes the risk of joint stiffness and facilitates faster recovery.

With the aging population, the incidence of osteoporotic proximal humerus fractures is projected to triple by 2030, placing a substantial burden on healthcare systems. RTSA, while costlier upfront than conservative or reconstructive surgical options, is a cost-effective solution in the long term due to its superior functional outcomes and reduced need for revision surgeries. Moreover, by restoring shoulder function, RTSA enables elderly patients to maintain independence, reducing the societal costs associated with long term care. Multiple meta-analyses [59–61] have highlighted the superior effectiveness of RTSA, showing lower complication and revision rates. These studies primarily focused on aspects such as geriatric fractures and their impact on postoperative clinical outcomes. Rossi et al. [62] reported no significant differences in clinical outcomes, complication rates, or revision rates between cemented and uncemented RTSA implants.

## Conclusion

Prosthetic treatment, particularly reverse total shoulder arthroplasty, has become the cornerstone for managing complex proximal humerus fractures in elderly patients. Its ability to restore function, relieve pain, and deliver durable outcomes makes it a superior choice compared to other treatment modalities. While challenges such as implant-related complications and surgical expertise remain, advancements in technology and techniques continue to enhance its efficacy. As the prevalence of these fractures rises, RTSA will play an increasingly critical role in preserving the quality of life for aging populations.

## Data Availability

No datasets were generated or analysed during the current study.
